# Effects of breed and colostrum replacer quantity on lymphocyte populations in the blood of Holstein and Holstein **×** Angus calves

**DOI:** 10.3168/jdsc.2025-0975

**Published:** 2026-04-09

**Authors:** M. Kovacs, H. McCarthy, T. Chapelain, L. Rostoll Cangiano, D.L. Renaud, M.A. Steele

**Affiliations:** 1 Department of Animal Biosciences, Animal Science and Nutrition, University of Guelph, Guelph, ON, Canada N1G 1Y2; 2 Department of Animal and Dairy Sciences, University of Wisconsin–Madison, Madison, WI 53706; 3 Department of Population Medicine, Ontario Veterinary College, University of Guelph, Guelph, ON, Canada N1G 1Y2

## Abstract

The objective of this study was to explore the effects of breed and colostrum replacer (CR) quantity fed at birth on lymphocyte populations in the peripheral blood of Holstein (H) and Holstein × Angus (C) crossbred calves. Male H (n = 10) and C (n = 10) calves were enrolled immediately after birth and randomly assigned to one of 2 quantities of CR: low (2.5 g of IgG/kg of birth BW; n = 4 for H and 6 for C) or high (5.0 g of IgG/kg of birth BW; n = 6 for H and 4 for C). Calves were tube fed CR meals at 0 and 12 h and then fed milk replacer at an average of 12% of BW for 21 d, followed by an average of 15% of BW until gradual weaning, which occurred between d 49 and 70. Additionally, the calves had ad libitum access to both fresh water and calf starter. Fecal consistency and respiratory health were evaluated twice daily to monitor health status. Blood was collected and analyzed at 24 h using radial immunodiffusion to evaluate serum IgG concentrations. Additional blood samples were collected on d 14, 35, and 84 of life for lymphocyte isolation and staining with monoclonal antibodies against gamma delta T cell receptor, WC1.1, WC1.2, IgM, CD21, and CD32 and analyzed by flow cytometry. Serum IgG was greater for the calves fed greater quantities of CR, but there were no differences between breeds. The proportion of lymphocytes among the peripheral blood mononuclear cells tended to be greater for H calves, whereas the proportion of IgM^+^ and IgM^+^CD21^+^ B cells among the total lymphocytes tended to be greater for calves fed higher quantities of CR. The results suggest that the quantity of CR may alter the lymphocyte profiles in peripheral blood of calves, highlighting the importance of colostrum on the development of the immune system in early life; however, the lymphocyte profile was not found to differ between H and C calves.

## Highlights

• Holstein and Holstein × Angus calves were fed low or high quantities of CR.
• The health of the calves was similar between breeds and CR groups.
• Higher CR quantity resulted in greater proportions of naïve B cells.
• Quantity of CR was associated with the development of certain lymphocyte subsets.

## Graphical Abstract

**Figure:**
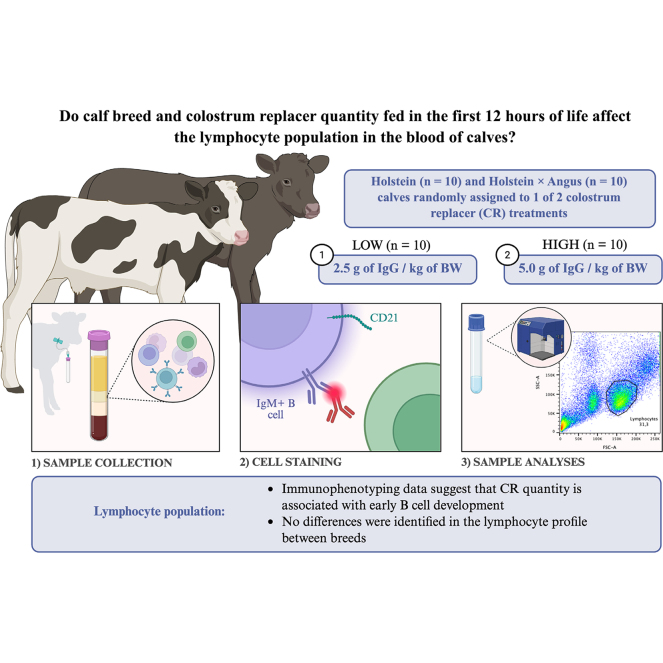
**Summary:**This study aimed to determine whether breed and colostrum replacer (CR) quantity fed in the first 12 hours of life affects lymphocyte populations in blood. Male Holstein (H; n = 10) and Holstein × Angus (C; n = 10) calves were fed CR at either low (2.5 g of immunoglobulin G [IgG]/kg of body weight; n = 4 for H and 6 for C) or high (5.0 g of IgG/kg of body weight; n = 6 for H and 4 for C) quantities at 0 and 12 hours of life and followed for an 84-day period at a commercial rearing facility. The study revealed that feeding higher quantities of CR was associated with a higher proportion of IgM^+^ and IgM^+^CD21^+^ B cells among the total lymphocytes, which were identified using flow cytometry. However, breed was not found to be associated with differences in the lymphocyte population in the peripheral blood of calves. Created with BioRender.com.

In recent years, dairy producers have been utilizing beef semen to inseminate a portion of their herd (Berry, 2021). This strategy is becoming particularly common in the North American dairy industry due to advancements in sexed semen and higher market values of Holstein × Angus crossbred calves compared with male Holstein calves (De Vries et al., 2008; Buczinski et al., 2021). Recent studies have reported that the resulting crossbred calves have reduced incidences and severity of disease during the first months of life compared with purebred Holsteins, notably a reduction in gastrointestinal disease (Moroz et al., 2025; Kovacs et al., 2026). However, the biological mechanisms underlying these differences in health outcomes are not fully understood.

Calves are particularly susceptible to diseases in early life because they have a limited capacity to mount robust immune responses and primarily rely on passive immunity acquired through colostrum for the first weeks of life (Chase et al., 2008). However, calves are born with lymphocytes that are important mediators of immune responses, aiding in pathogen clearance and the eventual development of effective immune responses during a period of heightened disease susceptibility (Montes and Reyes, 2013; Tizard, 2018). It is possible that variations in circulating lymphocyte populations among breeds may contribute to differences in immune responsiveness and subsequent health outcomes in calves.

Gamma delta T cells (**γδ T cells**) are a particularly important and abundant lymphocyte population found in neonatal calf circulation. These cells provide early protection against intracellular pathogens by rapidly responding to antigens in a major histocompatibility complex-independent manner, thereby linking the innate and adaptive immune systems when other immune components are not yet fully functional (Tizard, 2018; Guerra-Maupome et al., 2019). As passive immunity declines and active immunity begins to take over in the first weeks of life, B cells become increasingly prominent in circulation, serving an important role in conducting immune surveillance, and upon activation, producing antibodies and memory cells (Montes and Reyes, 2013). Although few studies to date have examined breed differences in lymphocyte populations in neonatal calves, differences have been reported in mature cattle. For example, Macêdo et al. (2013) observed lower proportions of T lymphocytes and greater proportions of B lymphocytes in the blood of Holsteins compared with Herford and Guzerá cattle, respectively. However, whether similar breed differences exist for lymphocyte populations during early life and contribute to differences in health remains unknown.

Early-life immune development is also strongly influenced by colostrum management. Beyond its role in providing passive immunity, research has also shown that colostrum influences the γδ T cell populations in calves (Krueger et al., 2016). Although exposure to an antigen-rich environment following birth is the primary trigger for γδ T cell expansion, a recent study demonstrated that the ingestion of colostrum is essential for maintaining the population and promoting early systemic immune programming (Cid de la Paz et al., 2025). However, these previous studies have been conducted exclusively in Holstein calves, leaving it unclear whether crossbred calves respond similarly to colostrum in terms of lymphocyte population development.

Therefore, investigating how colostrum management and breed influence lymphocyte profiles may help clarify underlying health differences between Holstein and Holstein × Angus calves. The objectives of this study were to compare γδ T and B cells in the peripheral blood of Holstein and Holstein × Angus calves, and to identify associations between the quantity of colostrum replacer (**CR**) fed and the lymphocyte populations. It was hypothesized that the proportion of γδ T and IgM B cells in circulation, and their functional subsets, would be greatest for Holstein × Angus calves fed greater quantities of CR.

All experimental procedures were approved by the University of Guelph's Animal Care Committee (AUP 4942) and all calves were enrolled between July and August 2023 at a commercial dairy farm in Southwestern Ontario. The dairy farm was selected due to its continuous calving surveillance and large herd size. This experiment was part of a larger study that involved a total of 48 calves which were randomly allocated to their respective treatments (Chapelain et al., 2024; McCarthy et al., 2026). However, due to logistic constraints, including labor availability and approval, only a subset of 20 calves from the final cohort were included in this experiment. The required biological replicate was determined using flow cytometry data from a previous study comparing colostrum-deprived and colostrum-fed calves (Krueger et al., 2016). Using an expected mean of 11.8% for the low colostrum group and 31.8% for the high colostrum group, SD of 6.48%, a power of 80%, and an alpha of 0.05, it was determined that 4 calves per group were needed to detect an absolute difference of 20% in γδ T cell proportions.

Male Holstein (**H**; n = 10) and Holstein × Angus (**C**; n = 10) calves from multiparous cows were enrolled in this study immediately after birth. Calves were removed from the dam within 30 min of calving, before suckling could occur, and weighed using a calibrated digital floor scale (Global Equipment Company Inc., ON, Canada). Only singlet calves weighing between 36 and 57 kg were included in the study. The calves were blocked by breed and birth BW and randomly assigned by the lead researcher to 1 of 2 CR groups using the RAND function in Microsoft Excel (version 2502, Microsoft Corp., WA). The low group (low; n = 4 for H and 6 for C) was provided 2 feedings of 2.5 g of IgG/kg of BW at 0 and 12 h of life (3.5 ± 0.44 L; 239 ± 29.6 g of IgG) and the high group (high; n = 6 for H and 4 for C) was provided 2 feedings of 5.0 g of IgG/kg of BW at 0 and 12 h of life (7.0 ± 0.66 L; 470 ± 44.7 g of IgG). Bovine-derived CR powder (Saskatoon Colostrum Company Ltd., Saskatoon, SK, Canada) was weighed out according to birth BW and mixed at 27.5% solids (27.0% IgG, 53.1% CP; 21.4% crude fat on a DM basis) with warm water (40°C). The first meal was fed within 1 h of birth and both CR meals were fed by one of 6 researchers using an esophageal tube feeder to minimize differences in feeding times and ensure all CR was ingested. The imbalance in group size resulted from logistical constraints related to sampling.

Between d 2 and 7 of life, calves were transported to a calf-rearing facility in Midwestern Ontario for the remainder of the study. Calves were fed milk replacer (24% CP, 20% fat on a DM basis) individually via a pail twice daily starting at 24 h of life. For the first 21 d of life, milk replacer was fed at an average of 12% of calf BW, and then increased to an average of 15% of calf BW until weaning. Milk replacer was mixed with warm water (42°C) to result in a final concentration of 130 g/L. Calves began the weaning process on d 49 and followed a gradual step-down approach: 11% of BW (d 49 to 56), 9% of BW (d 56 to 63), 4% of BW (d 63 to 70; one feeding per day), and complete weaning on d 70. Additionally, the calves had ad libitum access to fresh water and a common calf starter that was fed in individual troughs. Health examinations were conducted twice daily by one of 4 trained individuals, with the majority of health examinations carried out by a single observer that was blinded to CR group. Fecal consistency was evaluated on a 0-to-3 point scale according to Renaud et al. (2020), whereas respiratory health was evaluated using criteria established by Love et al. (2014). Antimicrobial, nonsteroidal anti-inflammatory drug (**NSAID**), and electrolyte therapies were administered to sick calves based on the health scoring protocol and veterinary consultation as described in Kovacs et al. (2026).

Blood was collected from all calves at 24 h after the first CR feeding to evaluate serum IgG and prior to morning feeding on d 14, 35, and 84 of life for flow cytometry using a jugular catheter and extension set. During sample collection and analyses, researchers were blinded to CR group. Blood samples at 24 h, which were taken from the catheter with a sterile syringe, were transferred into 10-mL serum Vacutainer tubes (Becton Dickinson, NJ), allowed to clot at room temperature and then centrifuged. Serum was stored immediately at −20°C and then shipped on ice to the Saskatoon Colostrum Company Ltd. Quality Assurance Laboratory to evaluate serum IgG concentrations using radial immunodiffusion, which served as an indicator of passive immunity status (Chelack et al., 1993; Chamorro et al., 2014). Blood taken on d 14, 35, and 84 was transferred into 10-mL K2 EDTA Vacutainers (Becton Dickinson, NJ) to evaluate the lymphocyte profile using flow cytometry analysis. Samples were transported to the laboratory (University of Guelph, ON, Canada) immediately after collection for peripheral blood mononuclear cell (**PBMC**) isolation, as described by Chattha et al. (2009). Briefly, samples were centrifuged at 1,200 × *g* for 15 min at 21°C, and the buffy coat was harvested and diluted with PBS (Sigma-Aldrich, MO). Histopaque-1077 (Sigma-Aldrich, MO) was layered below to create a density gradient, and samples were centrifuged to isolate PBMC. Cells were washed, erythrocytes lysed using sterile water, and the PBMC were resuspended in PBS.

For immunophenotyping, PBMC were stained using fluorochrome conjugated antibodies according to Cangiano et al. (2024). Briefly, panel 1 was used to stain γδ T cell subsets, and panel 2 was generated to stain naïve B cells (Table 1). Samples were run on a BD FACSCanto flow cytometer (BD Biosciences, CA) with 50,000 events per sample and analyzed using FlowJo 10.10 (FlowJo LLC, OR). Briefly, the lymphocytes and single cell populations were gated using forward scatter versus side scatter properties. The plots were then transformed into histograms and the immune cell subsets were identified based on fluorochrome expression. Gates were defined using fluorescent-minus-one controls and applied across all samples.

**Table 1:** Antibodies and respective fluorochromes used for flow cytometric analysis of peripheral blood^1^

Target population	Target receptor	Antibody clone	Fluorochrome	Source
γδ T cells	TCR-γ chain	GB21A	APC	WSU
	WC^+^	BAQ4A	AF 405	WSU
	WC1.1	CACTB32	RPE	WSU
	WC1.2	BAQ159A	AF 488	WSU
B cells	IgM	BM-23	AF 647	Sigma
	CD21	CC21	FITC	Bio-Rad
	CD32	CCG36	RPE	Bio-Rad

1 TCR = T cell receptor; WC = workshop cluster; CD = cluster of differentiation; APC = allophycocyanin; AF = Alexa Fluor; RPE = R-phycoerythrin; FITC = fluorescein isothiocyanate; WSU = Washington State University Monoclonal Antibody Center.

All statistical analyses were performed using SAS (version 9.4, SAS Institute Inc., Cary, NC). Descriptive statistics were generated for baseline dam and calf characteristics. The effects of breed and CR group on each lymphocyte subset were analyzed individually using generalized linear mixed models with beta distribution, accounting for repeated measures within calf over time. Flow cytometry data were modeled using a logit link function, and LSM are presented as back-transformed values. For all outcome variables, models included the fixed effects of breed, CR group, time (where applicable), and their interactions. Calf was treated as the experimental unit and included as a random effect. In addition, covariance structures were selected based on the lowest Akaike's information criterion. Spatial power was used for the lymphocyte profile as the interval between measurements were unequally spaced. Data are reported as LSM ± SEM, which represent marginal means averaged across all sampling time points. Significance was declared at *P* ≤ 0.05, and tendencies at 0.05 < *P* ≤ 0.10.

Baseline characteristics of the dam and calf are presented in Table 2. Mean birth BW (47.4 ± 5.48 kg; *P* = 0.98) and age at transport (4.4 ± 1.82 d; *P* = 0.61) did not differ between groups. Serum IgG was higher at 24 h for the calves fed the high quantities of CR (22.9 ± 3.26) compared with those fed lower quantities (14.7 ± 3.00; *P* < 0.01). All calves underwent a bout of diarrhea and received electrolyte therapy. Clinical signs of bovine respiratory disease were observed in 25% (1/4), 50% (3/6), 33% (2/6), and 50% (2/4) of calves in the H-low, C-low, H-high, and C-high groups, respectively. Consequently, 75% (3/4), 100% (4/4), 83% (5/6), and 100% (6/6) of calves in these groups received at least one NSAID treatment, whereas 75% (3/4), 100% (6/6), 67% (4/6), and 100% (4/4) received at least one antimicrobial treatment. Although we were not powered to evaluate clinical health, we did not find any significant differences between the groups.

**Table 2:** Baseline summary statistics (unadjusted) for Holstein (H) and Holstein × Angus (C) crossbred calves fed low (2.5 g of IgG/kg of BW) or high (5.0 g of IgG/kg of BW) quantities of colostrum replacer at 0 and 12 h of life

Parameter	H-low		C-low		H-high		C-high		*P*-value		
	Mean	SD	Mean	SD	Mean	SD	Mean	SD	Breed	Colostrum	Breed × colostrum
Dam parity	2.3	0.50	2.5	0.84	3.8	1.17	2.8	0.96	0.34	0.05	0.14
Birth BW	48.5	8.38	47.4	5.61	47.3	4.16	46.4	5.99	0.71	0.69	0.98
Serum IgG at 24 h^1^ (g/L)	13.9	2.06	15.3	3.57	23.4	3.86	22.2	2.41	0.96	<0.01	0.40
Age at transport	4.3	1.71	4.2	2.14	5.0	2.10	4.0	1.41	0.55	0.74	0.61

1 All calves achieved successful transfer of passive immunity.

The results of this study demonstrated that calves fed higher quantities of CR tended to have an increased proportion of naïve B cells (IgM^+^; low: 20.3 ± 0.02%; high: 25.1 ± 0.02%; *P* = 0.10) and had a greater proportion of CD21^+^ naïve B cells (IgM^+^CD21^+^; low: 14.0 ± 0.01; high: 18.3 ± 0.02; *P* = 0.05) within the lymphocyte population, regardless of breed (Table 3). When expressed on B cells, complement receptor 2 (CD21), an activating receptor, interacts with complement components C3d and C3bi and enhances B cell responses, indicating its importance in immune function (Tizard, 2018; Cangiano et al., 2024). In the present study, calves were fed CR rather than fresh maternal colostrum; therefore, because most maternal leukocytes are killed during the manufacturing process, the observed changes in lymphocyte profiles cannot be attributed to these cells (Chandler et al., 2023). We hypothesize that certain bioactive components found in CR, including lactoferrin, haptoglobin, and oligosaccharides, may play a role in the development and maintenance of certain lymphocyte subsets. Lactoferrin, a protein found within the whey fraction of CR, is absorbed into circulation and known to have immunomodulatory functions (Actor et al., 2009; Playford and Weiser, 2021). In murine cells in vitro, lactoferrin has been shown to increase complement 3 receptor expression, promoting B cell maturation (Actor et al., 2009). We therefore hypothesize that lactoferrin from CR may indirectly upregulate the proportion of peripheral blood B cells expressing IgM and CD21. However, future research is needed to better understand the direct role of these bioactives in colostrum on immune cells.

**Table 3:** Flow cytometric analysis of peripheral blood mononuclear cell populations on d 14, 35, and 84 d of age of Holstein (H) and Holstein × Angus (C) crossbred calves fed low (2.5 g of IgG/kg of BW) or high (5.0 g of IgG/kg of BW) quantities of colostrum replacer at 0 and 12 h of life (LSM ± SEM)

Population^4^ (%)	Treatment^1^				SEM	Day			SEM	*P-*value^2^, ^3^		
	H-low	C-low	H-high	C-high		14	35	84		Breed	Colostrum	Day
Lymphocytes^5^	27.5	18.9	19.1	17.9	0.03	18.7	21.2	22.1	0.03	0.10	0.13	0.70
γδ T cells	27.9	24.6	22.7	20.5	0.03	32.7	16.3	24.4	0.03	0.39	0.15	<0.01
Regulatory γδ T cells	5.7	5.7	4.6	5.3	0.01	6.3	3.2	7.3	0.01	0.72	0.37	<0.01
Cytotoxic γδ T cells	17.8	15.0	15.0	12.9	0.02	21.1	10.3	15.4	0.02	0.24	0.22	<0.01
γδT cells expressing												
WC1.1^+^	22.7	25.4	21.3	26.8	0.04	22.5	20.1	30.1	0.02	0.29	0.99	<0.01
WC1.2^+^	66.7	63.7	67.7	64.2	0.02	68.6	63.4	64.7	0.03	0.12	0.74	0.54
Lymphocytes^6^	23.2	18.4	19.7	18.6	0.02	19.7	16.7	23.8	0.03	0.10	0.38	0.12
Naïve B cells	22.1	18.7	24.7	25.6	0.03	15.1	21.2	34.4	0.02	0.62	0.10	<0.01
Naïve CD21^+^ B cells	16.0	12.1	16.8	19.8	0.02	10.3	15.6	24.6	0.02	0.68	0.05	<0.01
Naïve CD32^+^ B cells	20.4	17.8	22.7	23.8	0.03	13.4	20.1	32.8	0.02	0.74	0.15	<0.01
IgM^+^ cells expressing												
CD21^+^	76.6	70.9	71.6	79.4	0.07	72.4	76.4	75.4	0.05	0.85	0.78	0.67
CD32^+^	94.6	96.1	94.1	93.9	0.03	90.2	95.9	96.5	0.02	0.76	0.57	<0.01

1 n = 4 (H-low), 6 (C-low), 6 (H-high), 4 (C-high).

2 No breed × colostrum interactions were significant and therefore are not presented.

3 Significance declared at *P* ≤ 0.05 and tendencies at 0.05 < *P* ≤ 0.10.

4 Proportion of lymphocytes from peripheral blood mononuclear cells; proportion of gamma delta T cells (γδ TCR^+^) in the lymphocyte gate; proportion of regulatory (WC1.1^+^) and cytotoxic (WC1.2^+^) γδ T cells in the lymphocyte gate; proportion of WC1.1^+^ or WC1.2^+^ cells in the γδ T cell gate; proportion of naïve B cells (IgM^+^) in the lymphocyte gate; proportion of naïve CD21^+^ or CD32^+^ B cells in the lymphocyte gate; proportion of CD21^+^ or CD32^+^ cells in the naïve B cell gate.

5 Proportion of lymphocytes from peripheral blood mononuclear cells isolated for the γδ TCR^+^ panel.

6 Proportion of lymphocytes from peripheral blood mononuclear cells isolated for the IgM^+^ panel.

Although colostrum quantity did not affect γδ T cell populations (low: 26.2% ± 0.02%; high: 21.6% ± 0.02%; *P* = 0.15) in peripheral blood in the present study, previous studies have noted the role of colostrum in maintaining γδ T cells as the predominant lymphocyte subset in early life. Krueger et al. (2016) observed near doubling in the proportion of γδ T cells in the PBMC of Holstein calves fed CR compared with those deprived of colostrum, whereas Cid de la Paz et al. (2025) found that the proportion of γδ T cells markedly decreased in the first weeks of life for colostrum-deprived calves compared with those fed frozen colostrum. It is possible that feeding CR at quantities lower than those used for the low treatment group in the present study would have yielded results comparable to those reported for colostrum deprived calves.

In this study, there was a tendency (*P* = 0.10; Table 3) for H calves to have a higher proportion of lymphocytes among the mononuclear cells compared with C calves. Differences in the proportion of lymphocytes between breeds is consistent with previous studies across multiple species, including cattle and poultry (Macêdo et al., 2013; Bílková et al., 2017). However, it is important to note that lymphocytes were identified based on forward scatter and side scatter properties in FlowJo, which may not readily differentiate lymphocytes from monocytes. To enhance the specificity of immune cell phenotyping, future studies should incorporate specific markers to more accurately differentiate lymphocyte populations from monocytes, such as CD14, which are predominantly expressed on monocytes. This is a limitation of the study and may have implications on the analyses of IgM^+^ B cells and γδ T cells.

Breed did not affect the γδ T cell (H: 25.2% ± 0.02%; C: 22.5% ± 0.02%; *P* = 0.39; Table 3) or IgM^+^ B cell (H: 23.3% ± 0.02%; C: 22.0% ± 0.02%; *P* = 0.62) populations in the peripheral blood. Contrary to our findings, previous studies have noted differences in the lymphocyte profile among breeds, such as greater proportions of circulating B cells in Holsteins compared with Guzerá cattle and lower proportion in Brown Swiss and Herefords compared with Gir cattle (Macêdo et al., 2013). Moreover, breed differences in T lymphocytes have been reported in Holstein and Jersey calves undergoing immune challenges, perhaps suggesting that differences may be evident when phenotypic differences in health are present (Langel et al., 2016). It is also important to note that circulating lymphocytes represent only a small fraction of the total immune cell population. Therefore, future studies incorporating additional immune cell subsets, lymphoid tissues, and greater replication are warranted to better understand what may be contributing to differences in calf health between breeds.

An effect of day on most of the immune cells (*P* < 0.01; Table 3) was also observed, reflecting immune development as calves transition from reliance on maternal immunity to active immunity (Chase et al., 2008). Previous studies have reported similar trends, where the proportion of γδ T cells are the highest and most predominant after birth, followed by a gradual decrease in the first months, hence the inclusion of this marker in panel 1. However, the opposite is true for the proportion of B cells in peripheral blood with a shift toward a greater proportion over time (Chattha et al., 2009; Cangiano et al., 2024). These gradual changes are also accompanied by an increase in αβ T cell populations, including CD4^+^ helper and CD8^+^ cytotoxic T cells (Cortese, 2009). As these T cell markers were not included in the panel, changes in total and αβ T cell populations could not be directly assessed. Inclusion of these markers in future studies would allow for more comprehensive characterization of T cell development in H and C calves.

In conclusion, these results suggest that greater quantities of CR were associated with an increase in the proportion of B cells in peripheral blood irrespective of breed. However, future studies are needed to better understand which components of CR influence the lymphocyte populations.

## References

[bib1] Actor J.K., Hwang S.-A., Kruzel M.L. (2009). Lactoferrin as a natural immune modulator. Curr. Pharm. Des..

[bib2] Berry D.P. (2021). Invited review: Beef-on-dairy – The generation of crossbred beef × dairy cattle. J. Dairy Sci..

[bib3] Bílková B., Bainová Z., Janda J., Zita L., Vinkler M. (2017). Different breeds, different blood: Cytometric analysis of whole blood cellular composition in chicken breeds. Vet. Immunol. Immunopathol..

[bib4] Buczinski S., Fecteau G., Blouin L., Villettaz-Robichaud M. (2021). Factors affecting dairy calf price in auction markets in Québec, Canada: 2008–2019. J. Dairy Sci..

[bib5] Cangiano L.R., Lamers K., Olmeda M.F., Villot C., Hodgins D.C., Mallard B.A., Steele M.A. (2024). Developmental adaptations of γδ T cells and B cells in blood and intestinal mucosa from birth until weaning in Holstein bull calves. J. Dairy Sci..

[bib6] Chamorro M.F., Walz P.H., Haines D.M., Oassler T., Earleywine T., Palomares R.A., Riddell K.P., Galik P., Zhang Y., Givens M.D. (2014). Comparison of levels and duration of detection of antibodies to bovine viral diarrhea virus 1, bovine viral diarrhea virus 2, bovine respiratory syncytial virus, bovine herpesvirus 1, and bovine parainfluenza virus 3 in calves fed maternal colostrum or a colostrum-replacement product. Can. J. Vet. Res..

[bib7] Chandler T.L., Newman A., Cha J.E., Sipka A.S., Mann S. (2023). Leukocytes, microRNA, and complement activity in raw, heat-treated, and frozen colostrum and their dynamics as colostrum transitions to mature milk in dairy cows. J. Dairy Sci..

[bib8] Chapelain T., McCarthy H., Kovacs M., Innes D.J., Wood K.M., Duarte M., Renaud D., Steele M.A. (2024). Growth and health of Holstein and Holstein x Angus calves from birth to the end of the grower phase. J. Anim. Sci..

[bib9] Chase C.C.L., Hurley D.J., Reber A.J. (2008). Neonatal immune development in the calf and its impact on vaccine response. Vet. Clin. North Am. Food Anim. Pract..

[bib10] Chattha K.S., Firth M.A., Hodgins D.C., Shewen P.E. (2009). Age related variation in expression of CD21 and CD32 on bovine lymphocytes: A cross-sectional study. Vet. Immunol. Immunopathol..

[bib11] Chelack B.J., Morley P.S., Haines D.M. (1993). Evaluation of methods for dehydration of bovine colostrum for total replacement of normal colostrum in calves. Can. Vet. J..

[bib12] Cid de la Paz M., Viquez-Umana F., Mancheno M., Fernandez-Wallace T., Mantovani H.C., Cangiano L.R. (2025). Exploring the impacts of colostrum on systemic immune development in dairy calves. J. Dairy Sci..

[bib13] Cortese V.S. (2009). Neonatal immunology. Vet. Clin. North Am. Food Anim. Pract..

[bib14] De Vries A., Overton M., Fetrow J., Leslie K., Eicker S., Rogers G. (2008). Exploring the impact of sexed semen on the structure of the dairy industry. J. Dairy Sci..

[bib15] Guerra-Maupome M., Slate J.R., McGill J.L. (2019). Gamma delta T cell function in ruminants. Vet. Clin. North Am. Food Anim. Pract..

[bib16] Kovacs M., Renaud D.L., Keunen A., Steele M.A. (2026). Health and performance of Holstein versus dairy × beef calves: A retrospective single-cohort study. J. Dairy Sci..

[bib17] Krueger L.A., Beitz D.C., Humphrey S.B., Stabel J.R. (2016). Gamma delta T cells are early responders to *Mycobacterium avium* ssp. *paratuberculosis* in colostrum-replete Holstein calves. J. Dairy Sci..

[bib18] Langel S.N., Wark W.A., Garst S.N., James R.E., McGilliard M.L., Petersson-Wolfe C.S., Kanevsky-Mullarky I. (2016). Effect of feeding whole compared with cell-free colostrum on calf immune status: Vaccination response. J. Dairy Sci..

[bib19] Love W.J., Lehenbauer T.W., Kass P.H., Van Eenennaam A.L., Aly S.S. (2014). Development of a novel clinical scoring system for on-farm diagnosis of bovine respiratory disease in pre-weaned dairy calves. PeerJ.

[bib20] Macêdo A., Marciano A., Rocha L., Alves-Júnior J., Faria A., Bittar J., Araújo A., Santos R., Martins-Filho O. (2013). Comparative phenotypic profile of subpopulations of peripheral blood leukocytes in European (*Bos taurus taurus*) and Zebu cattle (*Bos taurus indicus*). Genet. Mol. Res..

[bib21] McCarthy H., Kirkup A., Kovacs M., Chapelain T., Leao J.M., Renaud D.L., Steele M.A. (2026). Impact of breed and colostrum quantity on immunoglobulin G kinetics in Holstein and Holstein-Angus crossbred bulls. JDS Commun..

[bib22] Montes R.L., Reyes L. (2013). B cells: Molecular biology, developmental origin and impact on the immune system. Cell Biology Research Progress.

[bib23] Moroz M.S., Martin C.C., Daros R.R. (2025). Health and growth performance during the pre-weaning phase of Angus × Holstein crossbred and Holstein calves managed under the same conditions. Dairy.

[bib24] Playford R.J., Weiser M.J. (2021). Bovine colostrum: Its constituents and uses. Nutrients.

[bib25] Renaud D.L., Buss L., Wilms J.N., Steele M.A. (2020). Technical note: Is fecal consistency scoring an accurate measure of fecal dry matter in dairy calves?. J. Dairy Sci..

[bib26] Tizard I. (2018). Veterinary Immunology. 10th ed..

